# Rethinking Clinical Trials in Age-Related Macular Degeneration: How AI-Based OCT Analysis Can Support Successful Outcomes

**DOI:** 10.3390/ph18030284

**Published:** 2025-02-20

**Authors:** Marie Louise Enzendorfer, Merle Tratnig-Frankl, Anna Eidenberger, Johannes Schrittwieser, Lukas Kuchernig, Ursula Schmidt-Erfurth

**Affiliations:** Laboratory for Ophthalmic Image Analysis, Department of Ophthalmology and Optometry, Medical University of Vienna, 1090 Vienna, Austria

**Keywords:** age-related macular degeneration, artificial-intelligence, optical coherence tomography, clinical endpoints

## Abstract

Age-related macular degeneration (AMD) is a leading cause of blindness in the developed world. Due to an aging population, its prevalence is expected to increase, making novel and optimized therapy options imperative. However, both late-stage forms of the disease, neovascular AMD (nAMD) and geographic atrophy (GA), exhibit considerable variability in disease progression and treatment response, complicating the evaluation of therapeutic efficacy and making it difficult to design clinical trials that are both inclusive and statistically robust. Traditional trial designs frequently rely on generalized endpoints that may not fully capture the nuanced benefits of treatment, particularly in diseases like GA, where functional improvements can be gradual or subtle. Artificial intelligence (AI) has the potential to address these issues by identifying novel, condition-specific biomarkers or endpoints, enabling precise patient stratification and improving recruitment strategies. By providing an overview of the advances and application of AI-based optical coherence tomography analysis in the context of AMD clinical trials, this review highlights the transformative potential of AI in optimizing clinical trial outcomes for patients with nAMD or GA secondary to AMD.

## 1. Introduction

Age-related macular degeneration (AMD) is the leading cause of irreversible visual impairment in individuals over 55 years in developed countries, affecting millions worldwide and imposing a substantial burden on healthcare systems [1,2]. Due to aging populations, the prevalence of AMD is expected to rise significantly, further exacerbating the challenge of managing this complex progressive disease. Late-stage AMD is classified into two distinct forms: neovascular AMD (nAMD), characterized by the growth of abnormal blood vessels, and geographic atrophy (GA), defined by the progressive loss of the retinal pigment epithelium (RPE), photoreceptors, and underlying choriocapillaris [3]. Both forms result in central vision loss, severely affecting patients’ ability to perform essential daily activities such as reading, driving, and recognizing faces [1].

Treatment advances over the past two decades have markedly improved outcomes in nAMD. Anti-vascular endothelial growth factor (VEGF) therapies effectively reduce vision loss by suppressing neovascularization and controlling fluid accumulation in the retina. However, the burden of frequent intravitreal injections and the variability in patient response underscore the need for more tailored therapeutic approaches [4,5]. In contrast, therapeutic options for GA have only recently emerged, with complement inhibitors like pegcetacoplan (Syfovre, Apellis) and avacincaptad pegol (Izervay, Astellas Pharma) gaining regulatory approval in the United States (U.S.) in 2023 [6,7]. While these therapies represent significant milestones, their modest impact on functional outcomes highlights the urgent need for improved strategies to preserve vision [8].

Optical coherence tomography (OCT) is the cornerstone of AMD diagnosis and monitoring, offering a non-invasive imaging modality with high-resolution and three-dimensional visualization of retinal structures. It plays a crucial role in identifying biomarkers of disease activity, such as fluid compartments in nAMD or layer integration in GA, as well as detecting subclinical changes that may predict progression from early stages of AMD to advanced disease [2,9]. Despite its strengths, the detailed interpretation of OCT volumes remains labor-intensive and subject to variability among graders, particularly in the context of large multicenter clinical trials. These limitations hinder the timely and standardized assessment of disease activity, emphasizing the need for innovative solutions.

Artificial intelligence (AI) has emerged as a transformative tool in AMD research, with the potential to address these challenges and revolutionize both clinical practice and research. AI consists of subfields including machine learning (ML), deep learning (DL), large language models (LLM), and many more [10]. ML is a subset of AI that acquires knowledge automatically from experience and can identify the features that were previously extracted by humans. DL is a subgroup of ML where artificial neural networks are organized in layers. In medical image analysis, a common type of DL termed convolutional neural networks (CNN) is most prominent. By adding more layers between the input layer and the output layer, the data can be processed in a more abstract way, resulting in increased effectiveness. The advantage of CNNs is that, in addition to classification, it can perform feature extraction without human intervention. This is therefore called unsupervised learning or end-to-end learning. While ML reaches its limit with the discriminative power of the selected features, the performance of DL increases with the amount of data it is presented [9,11].

In the context of AMD, AI-based algorithms, particularly those employing DL, excel at analyzing large and complex datasets, enabling precise and reproducible quantification of biomarkers such as retinal fluid volumes, RPE atrophy, and photoreceptor integrity [12]. This capability not only reduces interobserver variability, but also accelerates data analysis, making it feasible to incorporate more sophisticated endpoints in clinical trials [13]. Moreover, AI has demonstrated utility in predicting disease progression, stratifying patients based on their likelihood of therapeutic response, and exploring the intricate relationship between structural changes on OCT and visual function [14].

The integration of AI into clinical trial designs has profound implications for optimizing therapeutic development in AMD. By improving patient selection, AI can ensure that trials enroll individuals most likely to benefit from investigational treatments, thereby enhancing statistical power and reducing costs [15]. Additionally, AI-driven analyses of multimodal imaging data can uncover novel biomarkers and refine our understanding of AMD pathophysiology, paving the way for more targeted and effective interventions [9]. Finally, by bridging the gap between structural and functional assessments, AI offers a holistic approach to evaluating treatment efficacy, addressing both anatomical changes and their impact on patients’ quality of life [16,17].

This review explores the transformative role of AI in rethinking AMD clinical trials, emphasizing its potential to address unmet needs in therapeutic development and optimize outcomes for patients with nAMD and GA.

## 2. AI for Optimizing Clinical Trial Outcomes in Neovascular AMD

AMD is, per definition, a degenerative disease, but the development of macular neovascularization (MNV) may represent a serious complication [2]. In the active disease state, the neovascular network leaks, which results in pathological fluid accumulation, leading to acute and severe vision loss [18]. The management of nAMD has seen a breakthrough with VEGF inhibitor therapies, reducing blood and fluid leakage, thus helping patients maintain their visual acuity [19]. However, nAMD treatment trials show large variability in disease progression and treatment response among patients, with an estimated range of 10–50% of patients showing suboptimal or inadequate responses, depending on the study criteria used [20]. This is especially true in more long-term follow-up studies. AI can address these issues by enabling more precise patient stratification, identifying subgroups with shared disease characteristics or treatment responses through quantitative image analysis. Additionally, AI can help optimize trial design by predicting patient outcomes and by suggesting personalized endpoints that more accurately reflect meaningful improvements.

### 2.1. Clinical Endpoints and Monitoring of Neovascular AMD Using AI

OCT has emerged as the leading imaging modality for nAMD monitoring due to its ability to provide three-dimensional visualization of pathological fluid and retinal structure down to pixel-level precision [21]. Several studies have investigated the effect of fluid type as seen on OCT and the dichotomous presence/absence of fluid on visual function and anti-VEGF treatment outcomes. Differentiation is made between intraretinal fluid (IRF), subretinal fluid (SRF), and pigment epithelium detachment (PED). The negative impact of IRF on visual function has been shown frequently in numerous reports, while the effects of SRF and PED are still an ongoing debate [4,22,23,24,25,26].

An indirect parameter of exudation is the measurement of the average retinal thickness in the central 1 mm diameter of the Early Treatment Diabetic Retinopathy Study (ETDRS) grid. Central subfield thickness (CST) is a common measure for structural outcomes in clinical studies as it has been available since the early days of OCT [5]. CST can be either measured between the internal limiting membrane (inner border) and the RPE or the Bruch’s membrane (outer border) [5,27]. As macular fluids follow a distinct topographical distribution, CST is predominantly influenced by IRF and, depending on the definition, also by PED [28]. However, the majority of exudative fluid consists of SRF and PED, indicating that CST might not be fully representative of disease activity in nAMD [13]. This is further highlighted by the lack of correlation between CST with central IRF and SRF or CST with best-corrected visual acuity (BCVA) [29,30].

**Figure 1:**
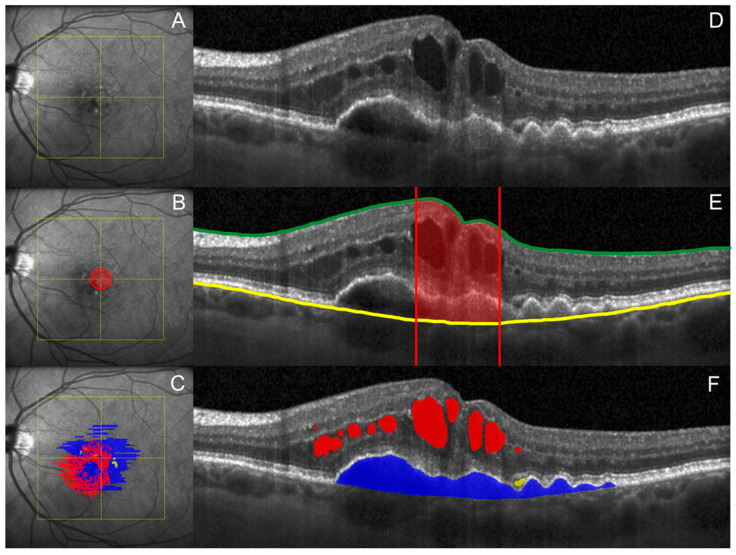
En face near-infrared reflectance image (**A**–**C**) and central B-scan (**D**–**F**). (**B**,**E**): Schematic illustration of CST, the mean thickness in the central 1 mm diameter of the ETDRS-grid (red) between the internal limiting membrane (green) and the Bruch’s membrane (yellow). (**C**,**F**): Example of automated segmentation of IRF (red), SRF (yellow) and PED (blue) using the Fluid Monitor (Version 2.5, RetInSight, Vienna, Austria). ETDRS = Early Treatment Diabetic Retinopathy Study, CST = central subfield thickness, IRF = intraretinal fluid, SRF = subretinal fluid, and PED = pigment epithelium detachment.

As CST is not an ideal indicator of exudation in nAMD, quantitative fluid measurement is required in order to allow for a more precise depiction of disease activity. Whereas manual segmentation is practically unfeasible and prone to errors, advancements in AI have driven the development of ML algorithms, enabling the objective and reliable quantification of fluid compartments on OCT, and thus offering a precise alternative to manual or qualitative assessment in real-time. How CST measurements compare with fluid segmentation is visualized in Figure 1.

Prior to clinical application of AI-based image segmentation methods, algorithms are typically evaluated based on the metric of the Dice Similarity Coefficient (DSC), which measures the spatial overlap between the automatically segmented region and the corresponding human reference annotation or ‘ground truth’. Additionally, receiver operating characteristic (ROC) curves and area under the curve (AUC) may be used to measure the discrimination ability of the algorithm for the specific task. For their successful use in clinical trials, algorithms must be consistent with or even superior to the ground truth of certified expert readings.

The pioneering Fluid Monitor (RetInSight, Vienna, Austria) is the first AI algorithm meeting the medical device regulation (MDR) 2017/745 requirements for class IIa medical devices as a clinical decision support system (CDSS) in ophthalmology [31]. It is based on a CNN and allows for pixel-wise quantification of IRF, SRF, and PED in the entire OCT volume [12]. Volume quantities are computed in nanoliters and visualized in a single report. By generating follow-up charts, it enables investigators to assess fluid dynamics and disease progression over time. In the U.S., it is currently available for research use only, with FDA approval for the application in the clinical routine being sought. RetinAI Discovery (RetinAI, Bern, Switzerland) is another European conformity CE-marked medical device incorporating a CNN that allows for fluid compartment quantification in OCT scans. In the U.S., the RetinAI module for fluid quantification may be used for research purposes only [32].

Retrospective analyses of pivotal trials examining anti-VEGF treatment outcomes in nAMD by leveraging AI-based biomarker segmentation of OCT volumes have yielded significant insights into the pathological implications of distinct fluid compartments and suggest refined assessments of treatment impact. These findings advance beyond the traditional dichotomous assessment of fluid presence or the conventional reliance on CST measurements and underscore the potential utility of novel biomarkers as refined clinical trial outcome measures. The application of automated fluid segmentation to the data of the HARBOR trial revealed characteristic resolution patterns of each fluid compartment after the first anti-VEGF injection, with IRF declining the greatest, followed by SRF, both of which remained low under treatment [13]. In contrast, the PED response was very limited, with no significant volume reduction during follow-up. Furthermore, the quantitative fluid analysis confirmed the detrimental impact of IRF on worse visual outcomes and a marginal positive correlation of SRF with BCVA [13]. A later analysis of the HARBOR data by Riedl et al. provided notable results. Considering only the follow-up period after the loading dose, SRF resolution was associated with a more pronounced functional improvement than IRF [33]. These differences between fluid compartments are not reflected by CST measurements, and thus currently often not taken into consideration when assessing treatment effects in clinical trials. Pawloff et al. [34] performed a comprehensive evaluation of the algorithm used for the above-mentioned analyses [12], using data from the HAWK and HARRIER trials. The evaluation demonstrated that the identification of fluid volumes using DL-based segmentation of retinal OCT scans reached high concordance with expert gradings performed by clinicians, efficiently achieving precise results while avoiding human inter-reader variability [34].

The FLUID study contributed to a shift in paradigm, suggesting that SRF, particularly when stable or minimal, may not need to be eliminated in the management of nAMD. In this study, visual acuity outcomes in an intensive treat-and-extend (T&E) arm, which aimed for complete resolution of qualitatively assessed IRF and SRF, were compared to outcomes in a relaxed T&E arm, which tolerated SRF unless it exceeded a threshold of 200 μm at the foveal center before extending treatment intervals. Originally, the study demonstrated that both treatment arms achieved comparable visual acuity after two years with fewer injections in the relaxed arm [4]. However, a post hoc analysis by Reiter et al. using AI-based pixel-wise fluid quantification revealed no significant quantitative differences in SRF or IRF between the two treatment arms at any study-specific time point, highlighting the more objective and precise fluid determination achieved using AI [35]. A further post hoc analysis investigating the impact of residual SRF volumes on treatment outcomes showed that interval extension despite residual SRF led to a significant increase of SRF volume and associated BCVA decline at the subsequent visit. However, this effect was measured only in the short term [36]. These analyses highlight the value of precise quantification of fluid compartment volumes made possible by AI for capturing true structure–function correlations, enabling a more suitable assessment of treatment efficacy.

The HAWK and HARRIER studies were large, randomized, phase III clinical trials designed to evaluate the efficacy, safety, and durability of brolucizumab compared to aflibercept, two anti-VEGF treatments for nAMD [37]. Post hoc automated fluid quantification applied to this large clinical trial dataset revealed that high volumes of IRF, SRF, and PED individually result in progressive vision loss. By measuring PED volumes quantitively, fluctuations within this compartment could be shown during the follow-up for as long as 96 weeks, reflecting the progressive nature within the subretinal fibrovascular network [38]. While a previous study has shown the efficacy of aflibercept in resolving PED, an analysis by Schmidt-Erfurth et. al. on the application of automated and precise volume quantification to the HAWK and HARRIER data highlighted the superior efficacy of brolucizumab in PED reduction, suggesting the higher sensitivity of AI-based quantification to subtle treatment differences [38,39]. This capacity may be useful, especially when the goal is the steady suppression of disease activity. Further post hoc analysis of HAWK by Ehlers et al. using automated ML-enhanced segmentation demonstrated that high volatility of exudative features, including SRF, during the maintenance phase of treatment was associated with photoreceptor attenuation and BCVA loss [40].

Investigations of the implications of structure–function correlations are crucial for optimizing treatment protocols and outcomes. Post hoc analyses of the Fight Retinal Blindness! (FRB!) registry data compared the impact of high versus low macular fluid volumes on structural and functional outcomes. IRF, SRF, and PED were automatically quantified on OCT using an AI-based tool [12]. As in the previous studies discussed, the unfavorable effect of IRF on BCVA outcomes was confirmed. Regarding SRF and PED, there was no significant difference between the high- and low-volume subgroups in BCVA outcomes, despite patients with high SRF and PED volumes receiving significantly more injections [41]. Further, Mares et al. investigated the FRB! study data on the association of AI-based fluid quantification with the structural integrity of the automatically segmented ellipsoid zone (EZ) layer, a structural biomarker for photoreceptor integrity [42,43]. High central IRF volume had the most severe negative impact on EZ thickness, while high SRF volumes showed no significant association with EZ integrity changes. Conversely, the high PED subgroup showed a significant association with both EZ thinning and loss [43]. With EZ integrity shown to be closely related to visual acuity, robust structure–function correlation assessments should contain both fluid volumes and information about the structural integrity of the neurosensory retina [40,44].

Given these findings, beyond the functional and conventional morphological endpoints typically used in pivotal trials, a detailed assessment of the treatment’s efficacy in terms of reliable and objective biomarker analysis has become essential. Such an approach allows for a more comprehensive characterization of available and emerging therapies, enhancing our understanding of how best to tailor treatment regimens and change the course of the disease to achieve optimal functional outcomes and personalized precision medicine. Two pioneering clinical trials (NCT04662944, NCT05093374) are investigating the impact of AI-assisted fluid quantification on patient treatment in outpatient clinics, however the results have not yet been published.

### 2.2. Leveraging AI for Patient Recruitment to Enhance nAMD Treatment Outcomes

Previously discussed analyses emphasized the importance of distinguishing between types of pathological fluid, as each fluid subtype exhibits distinct responses to anti-VEGF therapy. This differentiation is critical for tailoring treatment strategies and optimizing clinical trial outcomes. However, even before the onset of exudation, the classification of MNV subtypes may serve as a valuable indicator for identifying patients who are likely to derive the greatest benefit from treatment. Type 1 MNV involves neovascular growth beneath the RPE and is more strongly linked to SRF. Type 1 MNV requires more frequent injections but has a lower risk of developing GA and better long-term vision maintenance. Type 2 MNV arises from choroidal vessels breaking through the RPE into the neuroretina and typically responds faster to treatment due to its location above the RPE. However, it also carries a higher risk of fibrotic scarring [45,46], impacting visual outcomes [47]. Type 2 MNV tends to show higher levels of IRF compared to type 1 [48]. On the other hand, type 3 MNV originates from the retinal circulation, with neovascularization growing from the deep capillary plexus toward the outer retina [18]. This latter form of MNV greatly benefits from early detection and treatment, as delayed intervention can lead to worse outcomes and higher rates of GA. While validated AI tools for MNV classification are not yet available, promising advancements highlight their potential. A recent study tested a novel AI algorithm (aiMNV) capable of detecting and segmenting MNV in eyes with nAMD using OCT and OCT angiography [49]. The algorithm demonstrated high diagnostic accuracy, with 96.4% sensitivity and 98.3% specificity, and excellent segmentation performance. In the future, validated AI tools capable of accurately identifying MNV subtypes could support clinicians in optimizing treatment plans, improving outcomes, and reducing the risk of overtreatment or undertreatment.

Moreover, AI models have shown significant promise in predicting treatment outcomes and individual needs for patients with nAMD. AI methodologies using OCT imaging biomarkers have successfully predicted retreatment intervals and visual outcomes in T&E regimens. IRF and SRF volumes were identified as critical predictive markers, reinforcing the importance of quantitative OCT analysis [50]. Additionally, a DL architecture has shown an 84.6% accuracy in predicting short-term treatment responders versus non-responders, outperforming ophthalmologists [51]. Looking at the plethora of anti-VEGF therapies available today, identifying the best treatment for each patient is crucial. Such an approach could pave the way for precision medicine in AMD, ensuring that patients receive the most effective therapy based on their unique disease characteristics and response profiles. AI models, by integrating clinical, imaging, and treatment data, hold the potential to revolutionize this process, offering personalized care and optimizing outcomes for patients with nAMD.

## 3. AI for Optimizing Clinical Trial Outcomes in Geographic Atrophy

The non-neovascular late-stage form of AMD, termed geographic atrophy (GA), is characterized by the progressive and irreversible degeneration of photoreceptors, RPE, and the underlying choriocapillaris. The slow progression of this form of AMD means it often remains unnoticed and is only diagnosed at a stage where vision is already significantly impaired. Furthermore, GA encompasses a range of diverse and phenotypically distinct forms, with visual function primarily influenced by the extent of affected area relative to the fovea [52]. GA currently affects 5–10 million people worldwide and its prevalence is expected to increase [53]. It is thus of critical importance that there is investment to find solutions to slow down the cellular degeneration and find treatments that prevent vision loss in patients.

Recently, the first two therapies targeting GA secondary to AMD, pegcetacoplan and avacincaptad pegol, received regulatory approvals in the U.S. However, challenges persist in the clinical trial landscape for GA. While the U.S. Food and Drug Administration (FDA) approved pegcetacoplan, the European Medicines Agency (EMA) denied its marketing authorization in Europe, citing a lack of clinically meaningful benefits for patients [8]. This decision was primarily based on limited improvements observed in functional endpoints during phase III clinical trials [7]. These developments underscore the urgent need to re-evaluate clinical trial designs to facilitate the development and approval of therapies that demonstrate significant and measurable improvements for patients.

### 3.1. Structural Clinical Endpoints and Monitoring of GA Using AI

Traditionally, fundus autofluorescence (FAF) is used for detecting and monitoring GA, due to the high contrast visualization of lesions it provides. On FAF, lesions clearly differentiate from non-atrophic retinal tissues as dark hypofluorescent areas, caused by an absence of fluorophores in areas of RPE loss [54]. As a result, GA growth rate, as measured on FAF, is often used as a primary outcome measure in clinical trials. Despite the sharply demarcated areas, manual segmentation on FAF is often a time-consuming procedure. The RegionFinder software (Version 1.5.0, Heidelberg Engineering) provides a semiautomated segmentation method for GA area measurement on FAF, which is available to any clinic using Heidelberg Engineering FAF devices [55]. While the software allows for a more robust documentation of GA growth, it is important to note that this tool still requires manual input to adjust the delineations, still costing clinicians time and allowing for inter-observer variability. Various groups have proposed new AI methodologies that fully automate the segmentation on FAF and may soon be implemented for the more accurate assessment of GA in clinical trials [56,57,58].

However, it is important to note that as a progressive disease, GA not only affects the RPE, but also causes degeneration of the neurosensory layers, such as the photoreceptors [59]. Changes in these layers are not visualized in FAF images, but can be visualized using high-resolution three-dimensional images provided by OCT [60]. Today, OCT has become the gold standard for GA management. As OCT images provide pixel-level resolution of the individual neurosensory layers, GA appears as the loss of the RPE and overlying photoreceptor layer, resulting in areas of hypertransmission into the choroid [61]. Various approaches for the segmentation of GA lesions on OCT have been reported. Segmentation can be based on the en face OCT slabs [62], the RPE layer [63], photoreceptors [64], biomarkers associated with GA, such as hyper transmission [65], or a combination of features [66,67,68] to detect and quantify GA.

Clinical validation studies using such proposed AI algorithms are imperative prior to their use in clinical trials. A comprehensive evaluation of automated OCT monitoring of GA in comparison to the traditional FAF-based measurements used for clinical trial endpoints was performed as a post hoc analysis of the phase III OAKS and DERBY trials for pegcetacoplan by Mai et al. [69]. This study compared the area of photoreceptor loss and RPE loss on OCT, quantified using two previously validated algorithms [63,64,70], to assess reading-center manual FAF gradings. The study demonstrated that both cross-sectional and longitudinal assessments of GA progression showed a high correlation between manual FAF and automated OCT-based RPE measurements, highlighting the utility of automated OCT measurements as potential clinical endpoints. Similar results were reported by an independent group that investigated correlations of FAF measurements with automated GA segmentation on en face OCT images [71]. However, measurements of photoreceptor loss did not show a strong correlation with FAF-based GA measurements, highlighting that this may be a subclinical marker that is only achievable using AI-based OCT analysis [69]. The performance of the two algorithms for photoreceptor and RPE loss quantification compared to manually-performed human expert gradings was analyzed in two clinical validation studies [70,72].

The phase II and phase III clinical trials for the complement C3 inhibitor pegcetacoplan generated a large, high-quality multi-modal dataset and are thus an optimal source for post hoc analysis using novel AI algorithms [7,73]. These post hoc analyses shed light onto what may soon become standard clinical endpoint measurements and monitoring strategies for future clinical trials. A key finding, substantiated by work from various groups, is that the treatment effect of pegcetacoplan is more pronounced on the level of photoreceptor layer maintenance compared to the RPE, providing insight into the biological pathway and the effect of complement inhibition [72,74,75,76]. A topographic analysis by Riedl et al. used AI algorithms to generate topographic maps, presenting evidence that photoreceptor degeneration precedes RPE loss, contradicting the previous theory of photoreceptor loss as a result of RPE dysfunction [72]. This was also shown by Pfau et al. using a fully automated DL segmentation pipeline [14]. In particular, these analyses utilize automated segmentation of the EZ, a hyper-reflective band in the OCT corresponding to the ellipsoid of inner segments of photoreceptors [77]. As the inner segment ellipsoid is packed with mitochondria, the EZ as seen on OCT has become an important marker of photoreceptor health and function [78]. The post hoc analysis by Schmidt-Erfurth et al. of the OAKS and DERBY trials showed that the therapeutic impact on the EZ layer was notably more pronounced, with reductions of 53% and 46% in OAKS and 47% and 46% in DERBY for per month (PM) treatment and per every other month (PEOM), respectively, compared with sham at 24 months. In comparison, the reduction in RPE loss growth was shown to be 22% and 20% in OAKS and 27% and 21% in DERBY for PM and PEOM, respectively, at the same time point [76]. Also, Fu et al. reported a protective effect on both photoreceptors and RPE in their post hoc analysis of the OAKS and DERBY data, with the effect being more easily observed at the level of the photoreceptors [74]. Additionally, it is important to mention that EZ measurements have shown to be strongly associated with functional measures [79,80], making this a promising clinical endpoint that should also reflect functional changes.

**Figure 2:**
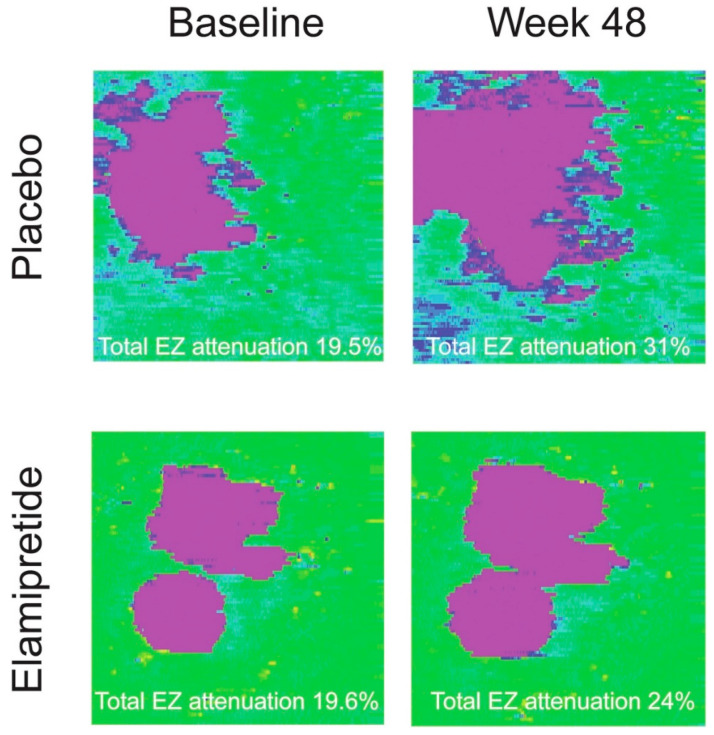
Representative EZ integrity maps from the ReCLAIM-2 trial evaluating elamipretide. Maps were chosen based on matching degrees of total EZ attenuation at baseline. These examples demonstrate increased EZ loss area growth in the placebo group compared to the treated group between baseline and week 48. Pink represents total EZ attenuation and blue represents partial EZ attenuation. Reproduced with permission from Ehlers et al., Ophthalmology Science; published by Elsevier, 2024 [81]. EZ = ellipsoid zone.

These analyses, only possible through precise AI-based quantifications, call for a rethinking of GA clinical trial design. This has recently been underlined by the acknowledgment of EZ loss as an outcome measurement in GA trials by the FDA. The phase II ReCLAIM-2 trial for elamipretide is the first prospective clinical trial to use measurements of EZ integrity as a clinical endpoint [81]. For the clinical study, EZ integrity was measured in a semi-automatic manner using a method previously described by Itoh et al. [82]. While elamipretide statistically did not meet the primary endpoints of mean change in low-luminance visual acuity (LLVA) and square root converted GA area on OCT, the therapy induced a 43% reduction in the mean progression from baseline in the percentage of total EZ attenuation and 47% reduction in the mean progression of percentage of partial EZ attenuation versus placebo at week 48. These results further highlight the higher sensitivity of measurements at the level of the photoreceptors to the progressive pathological changes associated with vision loss. The results also indicate that, as previously hypothesized, elamipretide supports photoreceptor preservation. Representative example cases are shown in Figure 2. As a result, measurements of EZ attenuation will serve as primary endpoint for the subsequent phase III studies [81].

These developments suggest the need for standardized regulatory-approved AI platforms that can be made available in clinics and retina practices worldwide. The GA Monitor (RetInSight, Vienna, Austria) consists of a composite algorithm that is able to segment and measure RPE and EZ loss and thickness, providing topographical maps that can be used for effective real-time decision-making. The tool has recently received MDR approval in Europe and has been approved for investigational use in the U.S. [83]. Extensively validated through post hoc studies [70,76,84], its real-time application in clinical trials is yet to be analyzed.

### 3.2. Improved Functional Endpoints in Geographic Atrophy Using AI

While structural imaging techniques like OCT provide valuable insights into anatomical changes in GA, functional outcome parameters are critical to assess the real-world impact of disease and to evaluate therapeutic efficacy. As mentioned earlier, the EMA’s decision to deny marketing authorization for pegcetacoplan in Europe emphasized the critical need for functional evidence in evaluating treatments’ impact on patients’ quality of life [8]. The data provided to the EMA suggested no statistically significant superiority in BCVA at 24 months in the intervention group [7].

The work of Pondorfer et al. explored the ability of various functional tests to differentiate between different stages of AMD, providing insights into their potential utility for generating meaningful data to support regulatory approval of emerging therapies. Their findings revealed that BCVA alone is insufficient to distinguish between early and intermediate stages of GA, as it remains relatively preserved until the disease involves the subfoveal region [85]. Sunness et al. presented data as early as 1997, showing varying visual impairments in patients with BCVA > 20/50, undetected by common visual testing [86]. More suitable functional tests include contrast sensitivity or LLVA, as contrast sensitivity is reduced early in GA patients due to impaired rod function and progressive photoreceptor degeneration, and reading speed due to its relevance to daily activities [87,88]. However, these functional parameters are also predominantly centered on foveal vision, which limits their ability to comprehensively evaluate GA. A significant proportion of patients show a parafoveal onset, sparing the fovea until late disease stages [89]. Consequently, measures focused solely on central retinal vision may fail to capture extrafoveal progression, scotoma formation, and the resultant quality of life impacts for patients. Extrafoveal GA progression commonly impairs the ability to read or recognize faces due to paracentral scotoma [59].

Microperimetry (MP) has emerged as a critical tool for addressing the limitations of conventional visual function tests. By mapping retinal sensitivity across the entire macula with precise fundus tracking, MP is able to detect localized changes in function and provides a more nuanced view of disease progression [90]. Several studies have conducted post hoc analyses, correlating structural imaging characteristics with functional MP measurements, using AI to superimpose the MP test points onto other imaging modalities, such as OCT [17,91]. Further, MP has enhanced our understanding of how and which structural pathologies lead to functional deficits. Using existing clinical datasets, Seeböck et al. have developed deep learning algorithms that are able to automatically predict a comprehensive retinal sensitivity map from an OCT volume [92].

**Figure 3:**
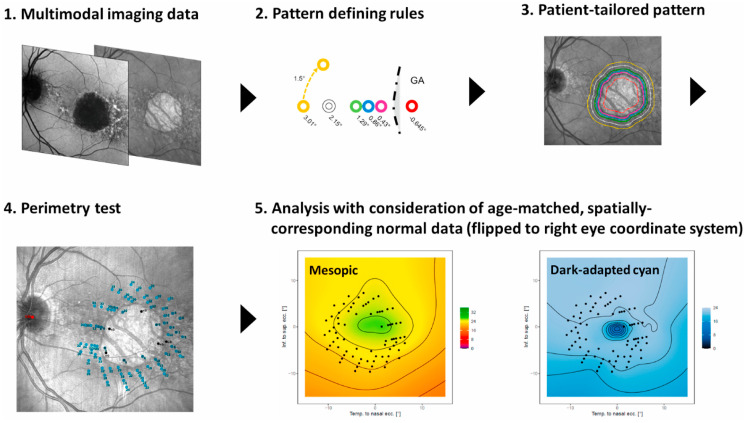
Example of an AI generated patient-tailored MP pattern shown by Pfau et al. (2021) [98]. Structural information from multimodal imaging data (1) is used to customize patterns. MP points are automatically placed on isohels surrounding the RPE loss border (2 and 3). The new pattern is projected onto the MP and subsequently tested (4). The different MP modalities, namely mesopic and dark-adapted cyan (5) are analyzed and compared to controls. Reproduced with permission from Pfau et al., Progress in Retinal and Eye Research; published by Elsevier, 2021 [98]. AI = artificial intelligence, MP = microperimetry, and RPE = retinal pigment epithelium.

Despite its advantages, standard MP patterns face limitations when applied to patients with advanced GA. Extensive scotomas often result in information loss of a significant number of test points, reducing the test’s overall informativeness. Additionally, areas of emerging atrophy may remain undetected if not actively targeted. Recent studies aim to advance MP technology through objective AI-based customizable test patterns, identifying areas at high risk for functional deficits, e.g., adjacent to atrophy or with thinned photoreceptors [93,94]. An example of an AI-generated patient tailored MP pattern is shown in Figure 3. By focusing on regions of residual function or areas at high risk of progression, MP now offers more precise and clinically relevant data. Studies by Wu et al. demonstrated how these algorithms improve both the reliability and predictive power of MP in assessing GA [16,95]. Further, automated targeted approaches enable a significant reduction in sample sizes for clinical trials due to decreased variance in retinal sensitivity.

Research by Wu et al., as well as Meleth et al., demonstrated that MP is capable of detecting subclinical functional impairments in retinal regions adjacent to RPE loss before structural changes become apparent on OCT [95,96,97]. AI-assisted post hoc analyses enable the correlation of reduced sensitivity with disruptions in the EZ and thinning of the RPE, thus bridging the gap between functional and structural assessments [14]. These findings underscore MP’s ability to provide actionable insights into disease mechanisms and therapeutic effects.

AI plays a transformative role in bridging the gap between structural imaging and functional assessments. By leveraging advanced ML algorithms, AI facilitates the integration of multimodal imaging data (e.g., OCT, FAF) with functional metrics such as MP, enabling more robust correlations. AI-assisted MP offers adaptive and personalized test patterns, targeting regions at high risk of functional decline, such as areas adjacent to GA lesions or those showing thinning of the EZ. This targeted approach enhances sensitivity in detecting subclinical changes, often preceding visible structural degeneration. For example, studies using AI to segment and quantify EZ integrity have shown strong correlations with visual outcomes and reading speed, directly linking photoreceptor preservation to patient-centric measures [80,99]. AI also improves the efficiency of functional assessments by automating large-scale data analysis and reducing interobserver variability. In GA trials, AI has been employed to analyze changes in retinal sensitivity across treatment and control groups, revealing significant functional preservation in targeted retinal regions. These insights reinforce the importance of combining structural and functional outcomes as dual endpoints for regulatory approval and real-world applications.

Functional endpoints are increasingly recognized as critical components of regulatory approval processes for GA therapies [100]. By enabling the AI-based detection of localized functional deficits and correlating these changes with structural biomarkers, MP provides a holistic understanding of disease progression and therapeutic impact. As the field advances, the integration of AI into clinical trial designs and regulatory frameworks will be instrumental in developing effective treatments for GA and getting regulatory approval. The adoption of targeted MP approaches, which focus on areas most susceptible to functional decline, promises to further refine these assessments and improve patient outcomes.

### 3.3. Leveraging AI for Patient Recruitment to Enhance GA Treatment Outcomes

Developing new drugs and bringing them to the market may be time-consuming and expensive as significant resources are dedicated to proving the safety and efficacy of novel treatments. A challenge in optimal trial design often lies in the recruitment of participants, where finding the eligible patients can cost time and money. Beyond monitoring GA lesion growth, methods to predict GA progression may be imperative for patient motivation, as well as for informed decision-making when it comes to patient inclusion. This is especially important in a slow-progressing, heterogenous disease such as GA, where progression patterns vary greatly between individuals. Also, for prognostic purposes, AI-based measurements of neurosensory layer thinning have shown to be of high value. In particular, it is the ratio between EZ loss and RPE loss that has been demonstrated to be highly predictive of GA progression rates [72,76]. This is also true in the context of treatment effects, whereby patients with a healthy photoreceptor layer, in other words a lower EZ to RPE loss ratio at baseline, are expected to see higher treatment benefits [76]. Stratifying patients by this personalized quantification of disease activity, rather than a population-based threshold, would allow for timely treatment decisions and improved patient outcomes.

Not only can AI tools be used for identifying ‘high-risk’ patients based on imaging features, AI-driven strategies may also aid in streamlining patient recruitment processes in clinical trials for GA by assessing large amounts of routine retinal images. By leveraging machine learning models, prescreening procedures can be automated, identifying eligible participants more rapidly and accurately [101]. This capability may not only alleviate recruitment challenges but may also ensure that diverse populations are represented in trials, thereby enhancing the generalizability of findings.

Various ML models to detect and classify GA have been reported on [102,103,104,105], but their value for specifically clinical trial recruitment remains understudied. A study conducted by researchers from Moorfields Eye Hospital and University College London demonstrated how AI provides an opportunity for automated prescreening for GA clinical trials [15]. The AI system evaluated TopCon OCT scans from 78,917 patients from a diverse population. The two-step approach features the segmentation of anatomic features and the use of a deep classification network to output probabilities for macular pathologies, including neovascularization, GA, and drusen. Patients had to have the right area and location of GA, as well as an absence of neovascularization to be counted as eligible. The study showcases the AI-based approach by using relevant inclusion criteria from the phase II HORIZON clinical study to shortlist patients. Analyses were then expanded to three addition trials including the phase III DERBY study of pegcetacoplan and the phase II GATHER2 study of avacincaptad pegol. The AI system successfully identified nearly twice as many eligible patients as the conventional screening method based on electronic health records. Differently to the conventional health record search, the AI system did not underrepresent certain ethnicities in patient selection [15].

## 4. Limitations of AI Integration for AMD Clinical Trials

The integration of AI into clinical trials for AMD still comes with several challenges and limitations. A major challenge lies in the quality and representativeness of the datasets used to train AI models. It must be remembered that the quality and accuracy of all ML-based tools depends on the quality of the training data. This makes ensuring training data represents diverse populations and is annotated in a standardized manner a pivotal issue. Many studies to date have relied on relatively small datasets, which raises concerns about the generalizability of findings to broader populations [106]. In addition, in the context of OCT based AI tools, a challenge lies in the development of cross-device algorithms. Most algorithms to date are specific to the device they are trained on (i.e., SPECTRALIS, Heidelberg Engineering). As such, clinics that are using different OCT devices may not benefit from the tools available.

Furthermore, the integration of AI technologies in clinical trials necessitates a robust governance framework to ensure the ethical use of AI while addressing potential biases in algorithmic decision-making. Ongoing research emphasizes the importance of inclusivity in data collection and the need for continuous training of healthcare providers in AI technologies to mitigate the risks associated with bias and inequity [107,108]. Additionally, respecting patient autonomy is a fundamental ethical principle in healthcare. This involves maintaining transparency in AI interactions and ensuring that patients are fully informed about the use of AI in clinical trials. Clear communication about how AI technologies will be applied in their treatment can enhance patient engagement and consent, aligning AI usage with ethical standards [109].

It is crucial for healthcare professionals to recognize the limitations of AI, even when using systems that have undergone extensive validation and received regulatory approval. The algorithms discussed within the scope of this review were designed and trained to perform previously defined tasks and may thus be insensitive to anomalies or outliers that may affect a patients disease progression and quality of life. Consequently, relying solely on AI could lead to missed pathological changes, resulting in false negatives or false positives that may significantly impact treatment decisions and patient outcomes. Therefore, AI should not be blindly trusted or seen as a replacement for clinical judgment; rather, it should be used as a supportive tool to aid, but not replace, the expertise of clinicians.

Future efforts need to focus on assessing the robustness of these AI systems in real-world scenarios, ensuring that they perform effectively across various imaging conditions and demographic groups. As the use of AI in clinical trials expands, it is essential to address ethical considerations and promote equitable access to these technologies. Inclusive data collection practices and regular equity audits of AI systems are vital for ensuring that underrepresented populations are adequately considered in research and treatment decisions. Developing ethical frameworks and maintaining transparency in AI operations will be crucial for fostering trust among stakeholders, including patients and healthcare providers.

## 5. Conclusions

This review underscores the transformative potential of AI in enhancing clinical outcomes for AMD. The application of AI-driven methods has demonstrated significant progress in automating biomarker analysis and standardizing evaluations, which are pivotal for large-scale clinical trials. For nAMD, AI-based segmentation of fluid compartments and dynamic disease monitoring have improved our understanding of disease activity and treatment response, facilitating more precise and personalized therapeutic strategies. Similarly, in GA, the deployment of AI has offered insights into the pathophysiology of disease progression and treatment efficacy. Emerging endpoints, such as EZ integrity and MP metrics, further illustrate the ability of AI to link structural and functional outcomes, promoting a multidimensional approach to disease assessment. The integration of AI in clinical trials may also optimize patient stratification, recruitment, and adaptive trial designs, ensuring that diverse populations are included and therapeutic benefits are maximized. However, challenges remain, particularly in ensuring the generalizability of AI models across diverse datasets, developing cross-device compatibility, and addressing ethical concerns, including bias and transparency in AI decision-making processes. Future research should prioritize the real-world validation of AI tools and the development of standardized regulatory frameworks to allow for the full integration of AI systems in clinical trial designs.

## Data Availability

No new data were created.

## Abbreviations

The following abbreviations are used in this manuscript:

AI	Artificial Intelligence
AMD	Age-related Macular Degeneration
BCVA	Best-Corrected Visual Acuity
CDSS	Clinical Decision Support System
CNN	Convolutional Neural Network
CST	Central Subfield Thickness
DL	Deep Learning
EMA	European Medicines Agency
ETDRS	Early Treatment Diabetic Retinopathy Study
EZ	Ellipsoid Zone
FAF	Fundus Autofluorescence
FDA	Food and Drug Administration
FRB!	Fight Retinal Blindness!
GA	Geographic Atrophy
IRF	Intraretinal Fluid
LLVA	Low-Luminance Visual Acuity
LLM	Large Language Models
MDR	Medical Device Regulation
ML	Machine Learning
MNV	Macular Neovascularization
MP	Microperimetry
nAMD	Neovascular Age-related Macular Degeneration
OCT	Optical Coherence Tomography
PED	Pigment Epithelium Detachment
RPE	Retinal Pigment Epithelium
SRF	Subretinal Fluid
T&E	Treat-and-Extend
U.S.	United States
VEGF	Vascular Endothelial Growth Factor
